# battery_xct_workflows: extracting quality metrics from X-ray computed tomography of Li-ion cells

**DOI:** 10.1016/j.mex.2026.103856

**Published:** 2026-03-09

**Authors:** Matthew P. Jones, Hamish T. Reid, Robert S. Young, Matt D.R. Kok, Francesco Iacoviello, James B. Robinson, Paul R. Shearing, Rhodri Jervis

**Affiliations:** a Electrochemical Innovation Lab, Department of Chemical Engineering, UCL, UK; b Advanced Propulsion Lab, Marshgate Building, UCL, UK; c Sention Technologies, Kings House, 9 - 10 Haymarket, London, UK; d The Faraday Institution, Harwell Science and Innovation Campus, Didcot, UK; e The ZERO Institute, The University of Oxford, Oxon, UK

**Keywords:** Batteries, Quality Assurance, X-ray CT, Python

## Abstract

battery_xct_workflows is an open, Python notebook-based method for deriving quantitative quality assurance (QA) metrics from X-ray computed tomography (XCT) of cylindrical Li-ion cells. The workflows target three recurring industrial QA questions: (i) are electrode overhang regions within design tolerance, (ii) is the canister geometry and alignment acceptable, and (iii) is the internal winding uniform and free from gross defects. The method combines preprocessing, segmentation (including optional pre-trained U-Net models), and metric calculation into a series of executable notebooks that can be run locally or via Binder using public example datasets.

By packaging data, code, models, and narrative explanations together, this method lowers the barrier to adopting XCT-based QA in both research and industrial settings. Users can reproduce the provided examples, adapt individual steps to their own scanners and cell formats, and extend the notebooks to new metrics while retaining a transparent audit trail.

## Graphical abstract

**Figure:**
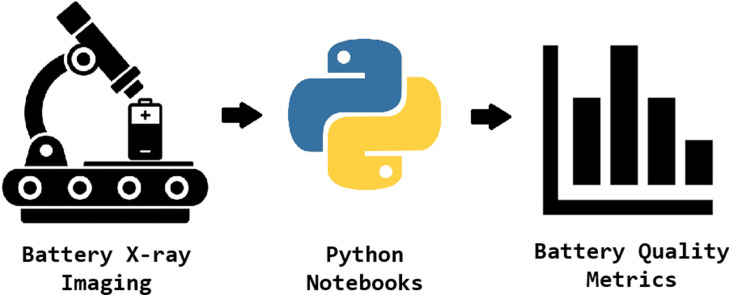

## Specifications table

**Table:** 

**Subject area**	Energy
**More specific subject area**	Batteries
**Name of your method**	Computational workflow for XCT-derived quality metrics in cylindrical Li-ion cells
**Name and reference of original method**	None
**Resource availability**	**Hardware:** 1. Industrial micro-focus CT scanner (e.g. Nikon 225 XTH). Software: 1. battery_xct_workflows GitHub repository (maintained); MPJ-Imaging/battery_xct_workflows: Open Jupyter Notebooks and Models for Lithium-Ion Battery QA using XCT 2. battery_xct_workflows Zenodo repository (archived) [1] **Models:** 1. Zenodo repository for pre-trained mini U-Net model for electrode overhang segmentations (archived) [2] **Example execution environment:** Binder links for running example notebooks in the cloud (no local install required) 1. Electrode Overhangs Metrics Calculation Notebook; https://mybinder.org/v2/gh/MPJ-Imaging/battery_xct_workflows/HEAD?labpath=notebooks/01_cylindrical_cell_overhangs.ipynb 2. Cell Canister Analysis Notebook; https://mybinder.org/v2/gh/MPJ-Imaging/battery_xct_workflows/HEAD?labpath=notebooks/02_cylindrical_cell_can.ipynb 3. Electrode Winding Quality Determination Notebook; https://mybinder.org/v2/gh/MPJ-Imaging/battery_xct_workflows/HEAD?labpath=notebooks/03_cylindrical_cell_electrode_winding.ipynb 4. Example Segmentation Notebook; https://mybinder.org/v2/gh/MPJ-Imaging/battery_xct_workflows/HEAD?labpath=notebooks/04_cylindrical_cell_segmentation.ipynb *Note on availability: we will endeavor to keep all links and online resources in this article active. If any hyperlinks change or become inactive, the underlying data, models, and notebooks will still be accessible via the archived Zenodo DOIs cited above.*

## Background

X-ray computed tomography (XCT) is now a routine tool for visualising the internal structures of lithium-ion (Li-ion) cells in three dimensions [3,4]. At the cell level, XCT is particularly valuable for identifying manufacturing and quality issues—such as electrode misalignment, canister deformation or winding defects—before they evolve into performance loss or safety incidents [5]. Subtle geometric faults can be difficult to detect by external inspection alone, but are readily visible in XCT images.

Despite this potential, there is still a gap between acquiring XCT volumes and obtaining quantitative, reproducible quality assurance (QA) metrics. In practice, many users stop at qualitative inspection of slices or volume renderings. Where quantitative analysis is performed, it often relies on *ad hoc* workflows, or proprietary software with limited transparency. This makes it hard to compare results across laboratories, reproduce published analyses, or systematically evaluate new QA strategies.

The method presented here responds to this gap by packaging battery-focused XCT analysis as a set of open, executable workflows rather than as a closed source software tool. The battery_xct_workflows notebooks demonstrate how to derive interpretable QA metrics for cylindrical Li-ion cells in three recurring scenarios: (i) quantifying electrode overhang using multiple geometric measures [[5], [6], [7]], (ii) measuring canister geometry and detecting dents via ellipse fitting, and (iii) assessing winding quality by transforming slices into polar coordinates and comparing them to an idealised spiral [8,9]. An additional notebook illustrates how both machine learning and classic computer vision approaches can be used to segment the electrode winding and overhang regions.

All workflows are implemented in Python using widely adopted scientific and machine-learning libraries, and are distributed as Jupyter notebooks bundled with example datasets and, where appropriate, segmentation masks and pre-trained models. They can be run locally or in the browser via Binder without specialised preprocessing or installation [10,11]. By coupling code, narrative explanation and data, the method is intended to make XCT-based QA analysis more accessible to battery researchers, engineers and quality specialists.

In this way, the methodology provides not only ready-to-use examples for common cylindrical cell QA tasks, but also adaptable templates that can be extended to other cell formats, additional metrics, and integration into testing pipelines. The overarching motivation is to lower the barrier to quantitative, transparent XCT analysis in battery science and manufacturing, and to support more reproducible methods across the community.

Whilst the individual operations used in these workflows (e.g., thresholding, morphology, ellipse fitting, distance transforms, and geometric measurements) are well established in the image analysis literature and many could be reproduced in graphical tools such as ImageJ/Fiji with appropriate plugins and macro scripting, the novel contribution of this MethodsX article is the publication of a first fully specified, reproducible, end-to-end workflow that couples narrative explanation, parameterised and versioned code, intermediate diagnostic validation outputs, and reference example data/models to derive a defined set of cylindrical-cell QA metrics; this packaging enables readers to repeat the method as published (including via a controlled Binder environment), and adapt the workflow while retaining a transparent audit trail.

## Method details

### Overview and implementation environment

The method is implemented as a set of executable Jupyter notebooks that operate on reconstructed XCT volumes of cylindrical Li-ion cells. All code is written in Python using standard scientific libraries (NumPy, SciPy, scikit-image, scikit-learn, Matplotlib, OpenCV, TensorFlow/Keras where required), and are distributed together with example datasets and pre-trained segmentation models [[12], [13], [14], [15], [16], [17], [18]]. Users can either (i) run the notebooks locally after cloning the repository and installing the dependencies, or (ii) launch them via a Binder link, which provides a preconfigured environment in the browser.

The workflow is structured as a single method with five main steps: (1) data acquisition and reconstruction, (2) segmentation of structures of interest, (3) electrode overhang metrics extraction, (4) canister quality analysis, and (5) electrode winding quality assessment. The notebooks are organised accordingly, and example data are provided so that each step can be executed end-to-end before being adapted to new cells or scanners. In addition to cell quality metrics, each notebook produces intermediate diagnostic outputs (e.g., overlay images of masks on raw slices, fitted geometry overlays, and residual/error maps) to allow users to validate segmentation quality and detect any failure of the analysis method on their own data early.

Step 1 Data acquisition and reconstruction

**Fig. 1:**
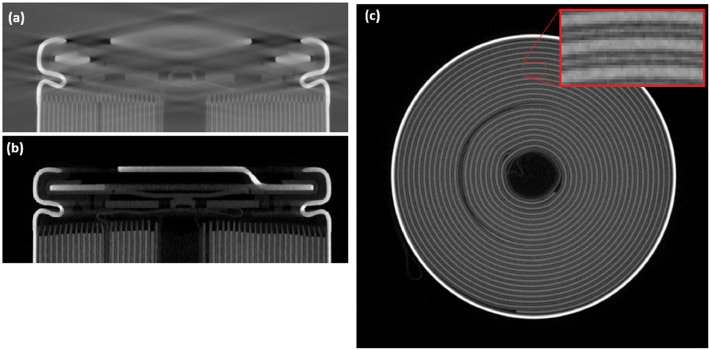
(a) example of poor data quality in a axial view slice due to cone beam artefacts, (b) an example axial view slice without cone beam artefacts, (c) the minimum recommended SNR in the radial view, the in-set image shows electrode layers are separated.

The method assumes XCT data acquired on a typical industrial CT system with a microfocus X-ray source (e.g., Nikon 225 XTH, Waygate Phoenix V|tome|x S240, Tescan Unitom HR) but is agnostic to specific hardware. The critical requirement is that the reconstructed volume has sufficient signal-to-noise ratio (SNR) and spatial resolution to detect the battery anode current collector (usually copper) and separate electrode layers, see Fig. 1*c*. In practice, an effective voxel size of approximately 30 µm is sufficient for standard 18650 and 21700 cylindrical cell formats when image quality is acceptable. Fig. 1 illustrates how acquisition and reconstruction quality directly affects the interpretability of XCT-derived QA metrics: panel (a) shows pronounced cone-beam artefacts in an axial view that distorts geometry and can bias segmentation and measurements, whereas panel (b) shows an axial slice without these artefacts suitable for downstream analysis. Panel (c) provides a practical minimum SNR criterion in the radial view, where separation of electrode layers (inset) indicates sufficient contrast and noise performance for reliable layer-resolved segmentation and downstream metric extraction.

A common acquisition pitfall is to use the full height of the detector to capture the entire cell in one scan. For tall objects this can lead to pronounced cone-beam artefacts in the reconstructed volume, as shown in Fig. 1*a*, which can compromise segmentation at the top and bottom of the cell. If cone-beam artefacts are problematic (and scanning the full cell is required), it is preferable to perform multiple overlapping scans at different heights along the cell, each with a smaller vertical field of view, and either analyse them separately or stitch them if required.

Any standard reconstruction algorithm can be used, including filtered backprojection (e.g., Feldkamp-Davis-Kress (FDK)) or iterative schemes. The downstream analysis is insensitive to the specific algorithm provided that the reconstruction is free from severe artefacts and preserves geometry. As a rule of thumb, the number of projections should be high enough to meet Nyquist sampling for the highest spatial frequency of interest in the cell; under-sampling will manifest as streaking and loss of fine detail in the electrode winding. Depending on the X-ray filter and acquisition parameters used, different beam hardening correction parameters will be required. Beam hardening correction is considered adequate when an intensity profile across a radial slice (through the central axis) shows no observable cupping when comparing the inner and outer electrode winding. The notebooks assume the reconstruction is exported as a 3D volume (e.g., a 16-bit TIFF stack or equivalent) with a known, homogeneous pixel spacing and that the cell axis is approximately aligned vertically. For reliable geometric measurements, cell tilt should be small; as a practical guide, a tilt below ∼1 degree is typically sufficient. A bespoke plastic 3D printed sample holder can be used to prevent tilt when the cell is mounted in the CT scanner.

Step 2 Segmentation of structures of interest

*Please see the example segmentations notebook (Notebook 4), available on the battery_xct_workflows Github repository, and executable in the cloud using Binder by following this link;* https://mybinder.org/v2/gh/MPJ-Imaging/battery_xct_workflows/HEAD?labpath=notebooks/04_cylindrical_cell_segmentation.ipynb

After reconstruction, the relevant volumes are loaded into Python using *tifffile* or an equivalent reader. We demonstrate two complementary segmentation strategies: (i) machine-learning-based segmentation using a pre-trained U-Net, and (ii) classical image processing using thresholding and binary morphology. Notebook 4 demonstrates both approaches. For the electrode overhang segmentation an open-source U-Net model is trained on axial slices. The model takes 2D slices as input and returns binary masks for the overhang; example code is provided for loading the weights, generating predictions, and post-processing the masks. Users who wish to apply ML segmentations to their own data can either use the supplied weights directly (if their contrast and resolution are similar) or fine-tune the model. Fine-tuning can span a range of user expertise. For experienced users, this may include modifying model hyperparameters (e.g., learning rate schedules, loss functions), expanding the training set, and increasing data augmentation to improve robustness across scanners and acquisition conditions. For less experienced users, fine-tuning can be performed by providing additional manually annotated or corrected masks and corresponding slices to the supplied training script, following the example workflow in the repository. Where Python-based model training is a barrier, users may alternatively employ GUI-based annotation/training front-ends to generate segmentation masks, provided that equivalent QC overlays are used to verify performance. The resulting overhang masks are then used as inputs to Step 3 (Notebook 1) for metric extraction.

For structures with strong contrast and simpler geometry, such as the metal canister and the bulk winding, a classical computer vision approach is often sufficient. In Notebook 4, these segmentations are performed with global thresholding combined with morphological operations (opening, closing, dilation/erosion) to remove noise and enforce connectivity. These steps are parameterised in the notebooks so that users can adjust thresholds and structuring elements for their own datasets. In all cases, the outputs are binary masks (or label images) that partition the volume into regions of interest: overhangs, electrode winding, canister, and background.

Step 3 Electrode overhang metrics extraction

*Please see the electrode overhangs metric notebook (Notebook 1), available on the battery_xct_workflows Github repository, and executable in the cloud using Binder by following this link;* https://mybinder.org/v2/gh/MPJ-Imaging/battery_xct_workflows/HEAD?labpath=notebooks/01_cylindrical_cell_overhangs.ipynb

**Fig. 2:**
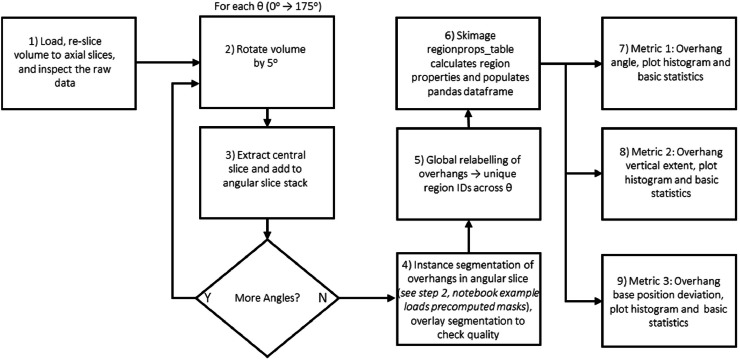
Flowchart summarizing the main processing stages in Notebook 1 for extracting cylindrical cell overhang metrics.

The overhang workflow quantifies the geometry of electrode overhangs of the cylindrical cell, and follows the steps illustrated in Fig. 2. The volume is first oriented so that the cell axis is vertical and cropped to a region encompassing the electrode stack near the top of the jelly roll. To characterise overhangs around the full circumference, the method approximates slicing normal to the winding by generating a series of angular slices at multiple rotation angles about the cell axis. For each angle, a central slice is extracted, producing a set of 2D images that sample different angular sectors. This angular slicing of the axial view is necessary to prevent warping where slicing is not normal to the electrode layer.

Using the ML-based overhang segmentation (implemented as in step 2), the overhang regions are identified in each angular slice. These masks are then used to compute a range of geometric descriptors, for example:

• Overhang angle or “deflection” relative to the radial direction, capturing the degree of misalignment.
• Vertical extent of overhanging regions.
• Overhang stacking height consistency.

**Fig. 3:**
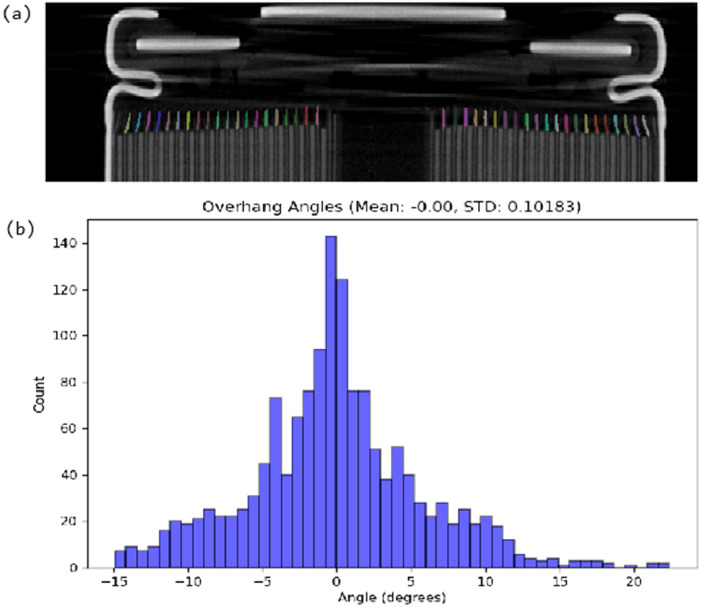
(a) Slice through a cylindrical cell tomogram with overhang masks overlayed. (b) Distribution of overhang deflection angles in the analysed cell. Overhang faults can lead to increased lithium plating and eventually short circuits.

Metrics are obtained using scikit-image’s regionprops_table and custom functions, and aggregated across all angles to produce distributions and summary statistics (means, maxima, and standard deviations). The notebook generates diagnostic plots such as histograms of overhang deflection, *see* Fig. 3, overhang length, and overlay images with masks on the original slices for validation. These results can easily be saved as tables (e.g., CSV) alongside these visualisations, providing a compact numerical summary of overhang behaviour for each cell.

Step 4 Canister quality analysis

*Please see the canister quality notebook (Notebook 2), available on the battery_xct_workflows Github repository, and executable in the cloud using Binder by following this link;* https://mybinder.org/v2/gh/MPJ-Imaging/battery_xct_workflows/HEAD?labpath=notebooks/02_cylindrical_cell_can.ipynb

**Fig. 4:**
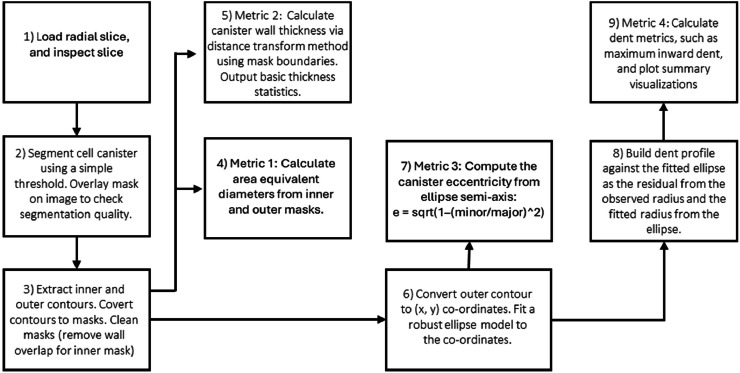
Flowchart summarizing the main processing stages in Notebook 2 for extracting cylindrical cell canister metrics.

The canister analysis workflow is illustrated in Fig. 4 and focuses on the geometry and integrity of the metal can enclosing the winding. A radial slice that intersects the full canister circumference is selected from the reconstructed volume. The canister material is segmented using grey-level thresholding and morphological clean-up to obtain a single, closed contour representing the inner or outer can wall.

From the segmented edge pixels, an ellipse is fitted to the canister cross-section using a least-squares procedure. The fitted ellipse parameters (centre, major and minor axes, and rotation) serve as a reference geometry from which several QA metrics are derived, for example:

• Outer/Inner Equivalent Diameters (area-equivalent circle diameters)
• Wall Thickness statistics via distance transform (mean / min / max / STD)
• Eccentricity from an ellipse fit to the outer boundary
• Denting (max inward deviation) relative to the fitted ellipse

**Fig. 5:**
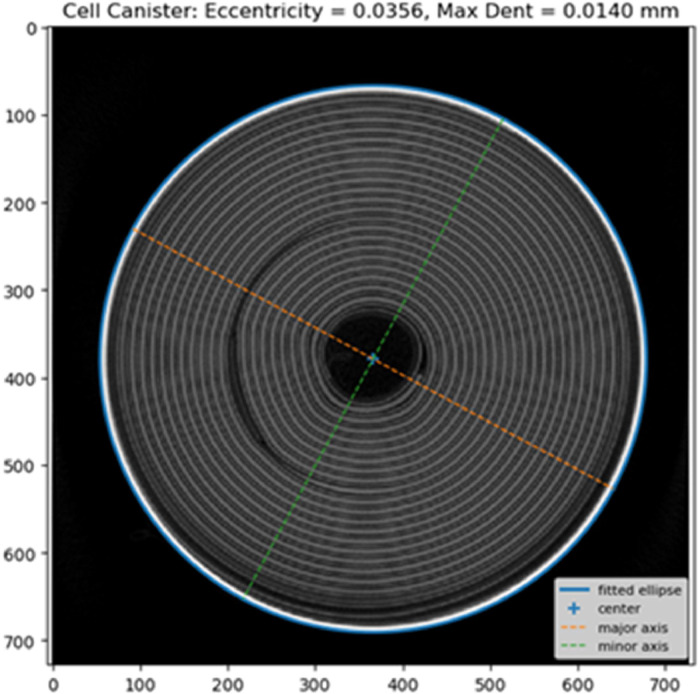
By fitting an ellipse to the canister we can measure a range of quality metrics. For example, the fitted ellipse is used to calculate canister eccentricity (a proxy for canister deformation) and is also used to detect and measure dents.

To detect dents or local deformations, the method computes the radial deviation of the actual canister boundary from the fitted ellipse. Localised negative deviations correspond to inward dents. The notebook creates visual overlays showing the fitted ellipse and prints canisters statistics (e.g., maximum dent depth, diameter, minimum wall thickness, etc.), *see* Fig. 5.

Step 5 Electrode winding quality assessment

*Please see the electrode winding quality notebook (Notebook 3), available on the battery_xct_workflows Github repository, and executable in the cloud using Binder by following this link;* https://mybinder.org/v2/gh/MPJ-Imaging/battery_xct_workflows/HEAD?labpath=notebooks/03_cylindrical_cell_electrode_winding.ipynb

**Fig. 6:**
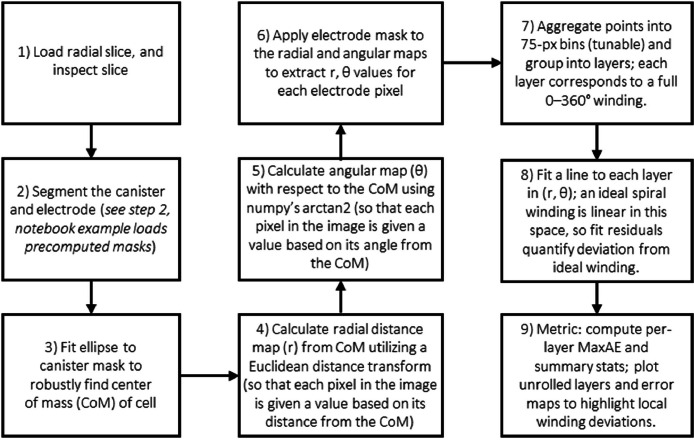
Flowchart summarizing the main processing stages in Notebook 3 for calculating electrode winding quality metrics.

**Fig. 7:**
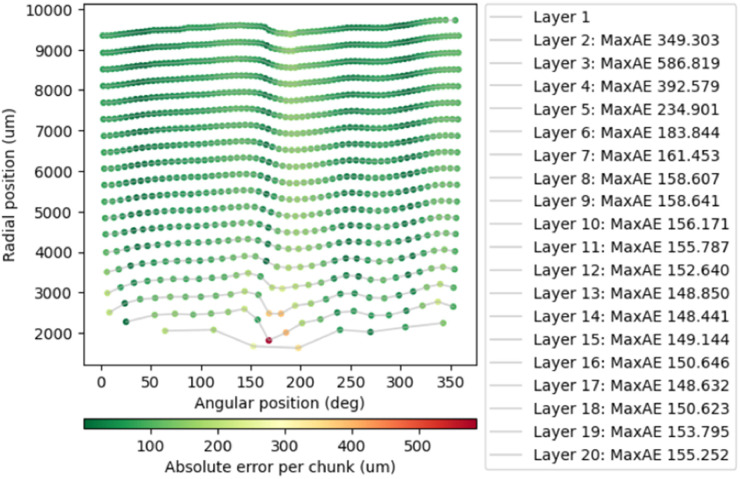
Cylindrical cell slice unrolled into polar coordinates and showing maximum absolute error (MaxAE) from a fitted spiral to detect buckling. In this case large buckles are detected in the central layers of the cell (layers 2 -4).

The winding quality workflow assesses how closely the electrode winding follows an ideal spiral and identifies regions of buckling; the general workflow is illustrated in Fig. 6. A representative radial slice through the electrode stack is selected, and the electrode is segmented (as in Notebook 4) into a binary mask, this mask is transformed into polar co-ordinates (r, θ) with respect to the centre of mass (CoM) of the cell. This was achieved with a Euclidean distance transform from the CoM to assign each electrode pixel a radial distance (r). Then, with array coordinates in image space, we compute the element-wise angle with respect to the CoM using np.arctan2 with offset coordinates to assign each electrode pixel a θ value. In this representation, one axis corresponds to angle around the cell centre, and the other to radial distance from the centre, effectively “unrolling” the winding into a polar image with clearly separated layers, *see* Fig. 7.

An Archimedean spiral is fitted to the observed layer positions to represent an ideal winding path. In polar coordinates, an Archimedean spiral is linear in θ: r(θ)=a+bθ, where a is the starting radius and b is the radial pitch. Therefore, after transforming the winding into polar space, we estimate the ideal path by fitting a straight lines (via linear regression) to each unrolled layer. Deviations between the observed layers and the fitted spiral are calculated along the winding, yielding metrics such as maximum absolute error and root-mean-square deviation.

These deviations are mapped back onto the unrolled image to produce heatmaps that highlight regions where the winding departs significantly from the ideal configuration, *see* figure 7. Large, localised deviations indicate electrode buckling.

## Method validation

Taken together, these five steps provide an end-to-end, notebook-based method for transforming reconstructed XCT volumes of cylindrical Li-ion cells into interpretable QA metrics for electrode overhangs, canister geometry, and winding quality. Each step can be run on the provided example data and then adapted to new cells by substituting input volumes, adjusting segmentation parameters, or retraining the segmentation model as needed. Validation of the method is easily demonstrated by running notebooks with the provided example data on the cloud using Binder. Additionally, in this article we provide illustrative intermediate outputs from the notebooks that demonstrate the resulting metrics.

To help ensure that the method continues to work as advertised, we include automated tests for both the core code and the notebooks. Simple checks are used to confirm that data-loading functions, model-building routines, and metric calculations behave as expected on small example inputs. In addition, key notebooks are executed automatically on a regular basis using a continuous integration service. This acts as a “smoke test” that the notebooks still run from start to finish with the current software environment, helping to catch issues early if dependencies change or the code is updated.

### Limitations

The current version of this method is tailored to cylindrical Li-ion cells and has been developed and tested using XCT datasets of standard cylindrical formats. Many of the ideas (e.g., overhang analysis, canister geometry, winding quality) do not directly transfer to pouch or prismatic cells, where the electrode architecture and failure modes differ. In its present form, the workflow should therefore be viewed as a template for cylindrical cell QA rather than a universal battery QA solution. A key aim for future versions is to extend these notebook workflows to other formats by developing equivalent metrics and visualizations for pouch and prismatic cells, and by providing representative example datasets for each case. In addition, consistent comparison of absolute metric values across laboratories is only meaningful when acquisition and reconstruction settings (voxel size, contrast, and artefact correction) are comparable; otherwise, the workflow is best used for within-site comparisons or for reporting metrics together with the key acquisition parameters.

The machine-learning component of the method is also limited by the training data currently available. The example U-net model for overhang segmentation has been trained on a relatively small, format-specific dataset, and its performance may degrade on cells with different chemistries, contrast conditions, or acquisition settings. Users are encouraged to treat this model as a reference implementation rather than a definitive solution. In future releases, we intend to expand the training dataset, retrain and benchmark the overhang model across a broader range of conditions, and include additional models (for example, for winding segmentation and denoising) with clear guidance on their expected scope of use.

Finally, the workflows assume that XCT data are of sufficient quality (resolution, noise level, and artefact control) for the derived metrics to be meaningful. Severe artefacts (e.g., cone-beam, ring, beam-hardening), under-sampling, motion blur, or very low phase contrast can reduce segmentation fidelity and bias geometric measurements. We provide fully worked example datasets that run end-to-end with default parameters and include intermediate diagnostic overlays (e.g., mask and fitted-geometry overlays) to help users judge whether their own data satisfy the method assumptions. However, applying the workflows to new scanners and protocols necessarily assumes practitioners can acquire reconstructions of comparable quality on their own system (e.g., by reusing established scan protocols or working with experienced operators); the workflow does not replace scanner-specific expertise in artefact mitigation. Future work will add more quantitative acceptance criteria and screening checks, expand guidance on common failure modes, and explore integration into automated QA pipelines, including uncertainty reporting on derived metrics.

## Ethics Statement

This work complied with all relevant ethical guidelines. It did not involve human participants, human data, or identifiable personal information; it did not involve animal experiments; and it did not involve data collected from social media platforms.

## CRediT author statement

**Matthew P. Jones**: Conceptualization, Methodology, Investigation, Software, Data Curation, Validity Tests, Visualization, Writing- Original draft preparation. **Hamish T. Reid**: Investigation. **Robert S. Young**: Investigation. **Francesco Iacoviello**: Investigation. **Matt D. R. Kok**: Conceptualization. **James B. Robinson**: Supervision. **Paul R. Shearing**: Supervision. **Rhodri Jervis:** Supervision, Writing- Reviewing and Editing.

## Related research article

None

## Declaration of competing interest

The authors declare the following financial interests/personal relationships which may be considered as potential competing interests:

Co-authors M.D.R.K., J.B.R. and P.R.S. are affiliated with Sention Technologies Limited, a company with potential commercial interest in applications related to this study. The company was not involved in the research presented.

## Data Availability

All code, example data, and models are publicly available via the maintained GitHub repository and archived Zenodo records (DOIs cited in the main text).

## References

[1] Jones, M.P., Reid, H.T., Young, R.S., Kok, M.D.R., Iacoviello, F., Robinson, J.B., Shearing, P.R., Jervis, R., 2025. battery_xct_workflows: Open Jupyter Notebooks for Lithium-Ion Battery QA using XCT. 10.5281/ZENODO.17625584.

[2] Jones, M.P., 2025. battery_xct_workflows: Example U-net model for overhang segmentation from CT of a battery. 10.5281/ZENODO.17624606.

[3] Heenan T.M.M., Tan C., Hack J., Brett D.J.L., Shearing P.R. (2019). Developments in X-ray tomography characterization for electrochemical devices. Mater. Today.

[4] Jiang Y., Tian A., Yan L., Du X., Yang L., Li L., Zhou J., Wang Q., Ruan S., He X., others (2024). X-ray computed tomography (CT) technology for detecting battery defects and revealing failure mechanisms. J. Electron. Mater..

[5] Attia P.M., Moch E., Herring P.K. (2025). Challenges and opportunities for high-quality battery production at scale. Nat. Commun..

[6] Roth T., Frank A., Oehler F., Graule A., Kücher S., Jossen A. (2024). Lithium plating at the cell edge induced by anode overhang during cycling in lithium-ion batteries: Part I. Modeling and mechanism. J. Electrochem. Soc..

[7] Yao X., Gorman D., Chatzopoulos G., Kubiak P., Sun W. (2025). Development of reference standards for metrological evaluation of battery electrode misalignment using X-ray computed tomography. J. Energy Storage.

[8] Kok M.D., Robinson J.B., Weaving J.S., Jnawali A., Pham M., Iacoviello F., Brett D.J., Shearing P.R. (2019). Virtual unrolling of spirally-wound lithium-ion cells for correlative degradation studies and predictive fault detection. Sustain. Energy Fuels.

[9] Sun W., Chen X., Kubiak P., López C.M. (2025). A new approach for health monitoring of cylindrical lithium-ion cells using X-ray computed tomography. J. Energy Storage.

[10] Jupyter P., Bussonnier M., Forde J., Freeman J., Granger B., Head T., Holdgraf C., Kelley K., Nalvarte G., Osheroff A., Pacer M., Panda Y., Perez F., Ragan-Kelley B., Willing C., Fitzjohn S., others (2018). Proceedings of the 17th Python in Science Conference.

[11] Kluyver T., Ragan-Kelley B., Pérez F., Granger B., Bussonnier M., Frederic J., Kelley K., Hamrick J., Grout J., Corlay S., Ivanov P., Avila D., Abdalla S., Willing C., Loizides F., Schmidt B., others (2016). Positioning and Power in Academic Publishing: Players, Agents and Agendas.

[12] Abadi, M., Agarwal, A., Barham, P., Brevdo, E., Chen, Z., Citro, C., Corrado, G.S., Davis, A., Dean, J., Devin, M., Ghemawat, S., Goodfellow, I., Harp, A., Irving, G., Isard, M., Jia, Y., Jozefowicz, R., Kaiser, L., Kudlur, M., Levenberg, J., Mané, D., Monga, R., Moore, S., Murray, D., Olah, C., Schuster, M., Shlens, J., Steiner, B., Sutskever, I., Talwar, K., Tucker, P., Vanhoucke, V., Vasudevan, V., Viégas, F., Vinyals, O., Warden, P., Wattenberg, M., Wicke, M., Yu, Y., Zheng, X., 2015. TensorFlow: Large-scale machine learning on heterogeneous systems.

[13] Bradski G. (2000). The OpenCV library. Dr Dobbs J. Softw. Tools..

[14] Harris C.R., Millman K.J., van der Walt S.J., Gommers R., Virtanen P., Cournapeau D., Wieser E., Taylor J., Berg S., Smith N.J., others (2020). Array programming with NumPy. Nature.

[15] Hunter J.D. (2007). Matplotlib: A 2D graphics environment. Comput. Sci. Eng..

[16] Pedregosa F., Varoquaux G., Gramfort A., Michel V., Thirion B., Grisel O., Blondel M., Prettenhofer P., Weiss R., Dubourg V., Vanderplas J., Passos A., Cournapeau D., Brucher M., Perrot M., Duchesnay É. (2011). Scikit-learn: Machine learning in Python. J. Mach. Learn. Res..

[17] van der Walt S., Schönberger J.L., Nunez-Iglesias J., Boulogne F., Warner J.D., Yager N., Gouillart E., Yu T., contributors, scikit-image (2014). scikit-image: image processing in Python. PeerJ.

[18] Virtanen P., Gommers R., Oliphant T.E., Haberland M., Reddy T., Cournapeau D., Burovski E., Peterson P., Weckesser W., Bright J., others (2020). SciPy 1.0: Fundamental algorithms for scientific computing in python. Nat. Methods.

